# Molecular and histological characteristics of bovine caudal nucleus pulposus by combined changes in hydrostatic and osmotic pressures in vitro

**DOI:** 10.1002/jor.24188

**Published:** 2019-01-03

**Authors:** Shuichi Mizuno, Kaori Kashiwa, James D. Kang

**Affiliations:** ^1^ Department of Orthopedic Surgery Brigham and Women's Hospital and Harvard Medical School 75 Francis Street Boston Massachusetts 02115

**Keywords:** intervertebral disc, mechanobiology, hydrostatic pressure, osmotic pressure

## Abstract

Intervertebral disc degeneration is ubiquitous among aging patients, and altered matrix homeostasis is one of the key features of this condition. Physicochemical stresses have a significant impact on matrix homeostasis as they lead to progressive degeneration and may be associated with spinal pain and dysfunction. Thus, it is important to understand the cellular and matrix characteristics of nucleus pulposus in response to these stresses, which include hydrostatic and osmotic pressures during alternate loading conditions. We hypothesized that a combination of changes in hydrostatic pressure and in osmotic pressure that mimic normal, daily spinal stress would stimulate anabolic function, whereas a non‐realistic combination of those stresses would stimulate catabolic function in nucleus pulposus cells. We examined the effects of these combined stresses, represented by 12 systematic conditions, on the metabolic activities of enzymatically isolated bovine caudal nucleus pulposus in vitro. We measured the gene expression of extracellular matrix (ECM) molecules and proliferating cell nuclear antigen (PCNA) and evaluated the quality of the matrix and the capability of cell proliferation immunohistologically. Combined cyclic hydrostatic pressure at 0.5 MPa, 0.5 Hz, and high osmotic pressure at 450 mOsm upregulated the aggrecan core protein and collagen type‐II gene expression significantly (*p* < 0.05), and showed trends of upregulation of chondroitin sulfate N‐acetylgalactosaminyltransferase 1, matrix metalloproteinase‐13, and PCNA. ECM, however, contained empty spaces at a high osmotic pressure with and without hydrostatic pressure. Since ECM has highly specialized physicochemical properties, homeostasis should involve not only phenotypic cellular behavior but also turnover of ECM. © 2018 The Authors. *Journal of Orthopaedic Research*® Published by Wiley Periodicals, Inc. on behalf of Orthopaedic Research Society. J Orthop Res 37:466–476, 2019.

The intervertebral disc (IVD) is composed of highly negatively charged amorphous extracellular matrix (ECM) in nucleus pulposus (NP) and abundant interstitial water.^1^, ^2^ Spinal motions in upright positions (e.g., lateral bending, flexion, and extension) and off‐loading in a recumbent position creates changes in physicochemical stresses momentarily and in daily cycles.^3^ These motions alter hydrostatic pressure (HP) and osmotic pressure (OP) during alternate loading stresses, and the degeneration of the IVD alters the quality of ECM and ultimately causes change in swelling pressure.^4^, ^5^, ^6^, ^7^, ^8^ Therefore, degeneration, regeneration, and homeostasis are encountered by changes in these stresses.^9^, ^10^, ^11^, ^12^, ^13^, ^14^ Recently, the mechanisms of IVD degeneration have been studied using conventional cell or explant culture models.^15^, ^16^, ^17^, ^18^, ^19^ These studies were limited in their ability to reproduce metabolism in IVD exposed to changes in physicochemical stresses.^20^, ^21^, ^22^, ^23^ A thorough understanding of how these stress variables affect matrix metabolisms will fill a large gap that exists in the field of IVD homeostasis and ultimately give a new direction to the development of biological treatments for IVD degeneration.^24^ Thus, our objective was to recapitulate these stresses in vitro and to clarify the effects of combined changes in OP and HP on metabolic functions in NP cells. We hypothesized that a combination of changes in HP (ΔHP) at high OP (HOP) mimicking daily spinal stress within a normal NP would maintain homeostasis in the NP. ΔHP at low OP (LOP) mimicking daily spinal stress within a degenerated NP would decrease anabolic activity or activate catabolic activity in the NP. In order to test this hypothesis, we systematically compared the interactions of combined ΔHP and ΔOP in three groups with a set of counter culture conditions: Group A) Interactions of combined HP and HOP, Group B) Interactions of combined ΔHP and ΔOP, and Group C) Interactions of simultaneous ΔHP and ΔOP (Fig. 1). We applied these ΔHP and ΔOP using a HP/perfusion culture system and semipermeable membrane pouches (Fig. 2). These systems allowed cyclic HP with constant medium replenishment to incubate suspended NP cells/clusters with various conditions for HP and OP of the medium. We evaluated the metabolic activity of NP cells by examining the gene expression of ECM molecules and proliferating cell nuclear antigen (PCNA), as well as the quality of ECM molecules and cell proliferation immunohistologically.

**Figure 1:**
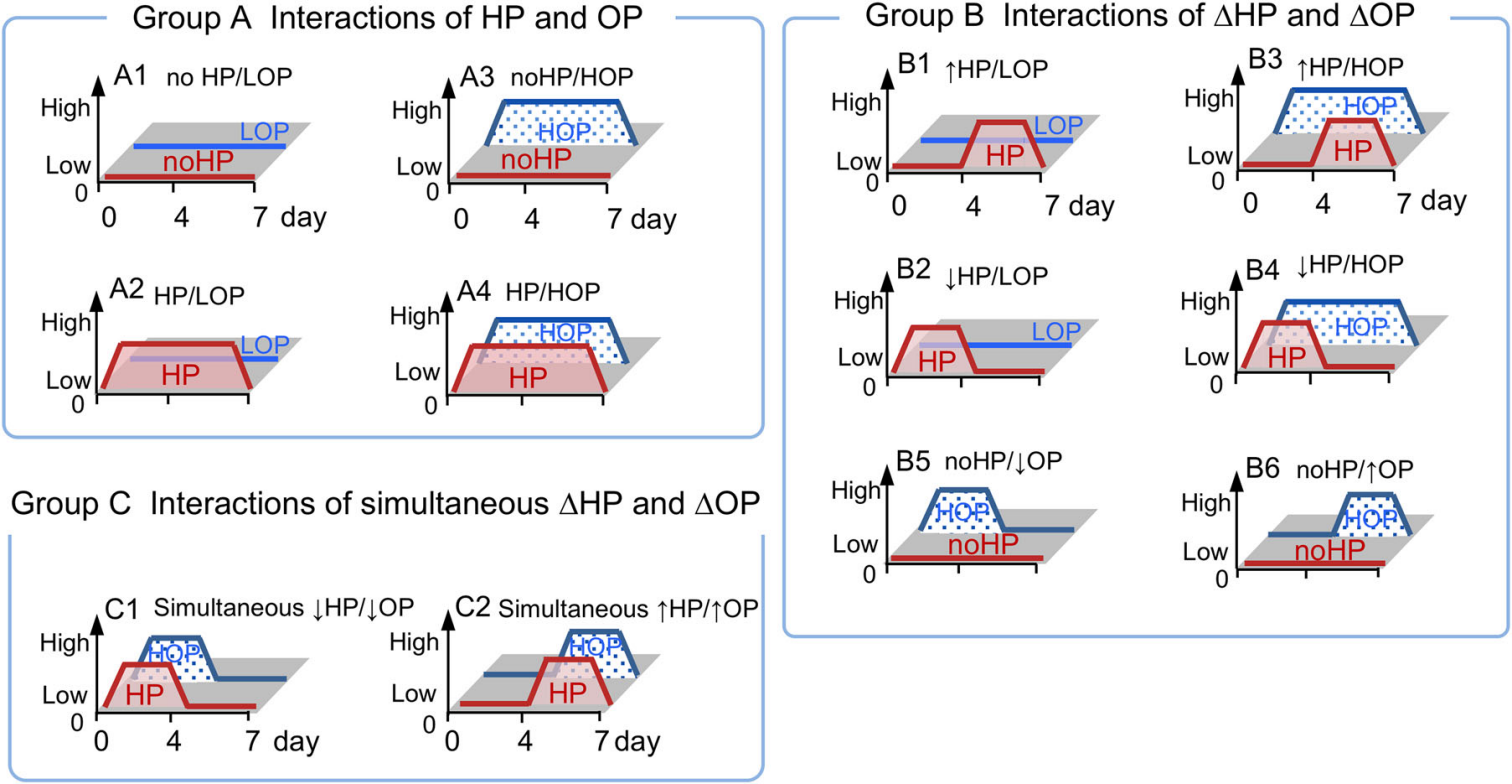
Systematic culture conditions to compare interactions of changes in in hydrostatic pressure and osmotic pressure. no HP: atmospheric pressure. HP: cyclic hydrostatic pressure 0–0.5 MPa, 0.5 Hz. LOP: low osmotic pressure (320 mOsm). HOP: high osmotic pressure (450 mOsm).

**Figure 2:**
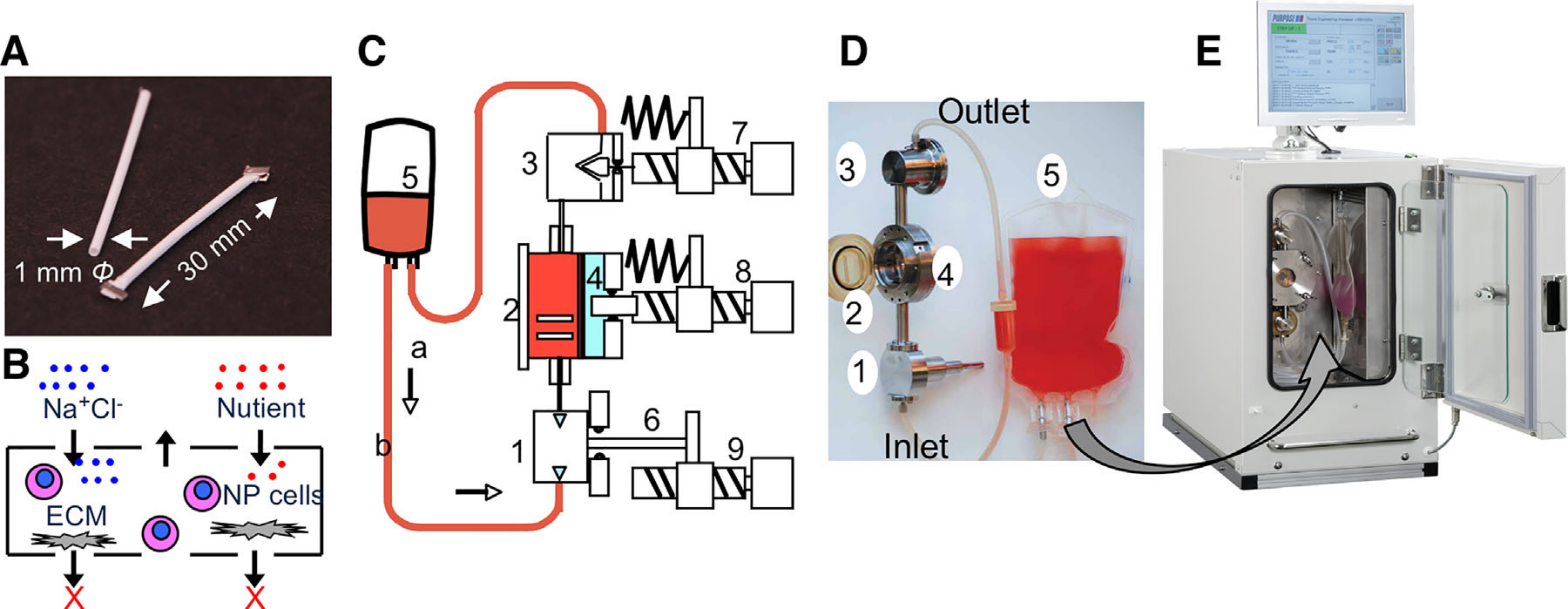
A semipermeable membrane pouch culture module and HP/perfusion culture system. (A) A semipermeable membrane pouch for enclosing NP cells/clusters. The pouch was made of polyvinylidene difluoride (1.0 mm in diameter, 1.2 mm outer diameter, 30 mm in length, 500 KD MWCO). (B) A diagram of the rationale of a semipermeable membrane pouch. Larger molecules for example, ECM is retained within the pouch, whereas smaller molecules (<500 kD) in‐ and out‐fluxes through the membrane. (C) A diagram of HP/perfusion culture module. The culture module has three components: (1) a pump unit, (2) a pressure‐proof culture chamber unit, and (3) a backpressure control unit. The culture unit has a flexible plastic film (tetrafluoroethylene perfluoroalkyl vinyl ether) that separates the culture chamber from (4) the adjacent water compression chamber. Water in the compression chamber was compressed with 8) an actuator driven piston, and HP was transduced to the medium through the flexible plastic film. HP in the medium and in the water chamber was equivalent so that we could monitor the HP within the water compression chamber with a pressure sensor. Each unit is connected with pressure‐proof unions. Outlet of a backpressure control unit and inlet of a medium perfusion pump unit were connected to a medium bag by silicon tubing allowing gas exchange but completely closed to air. 5) Culture medium is kept in a clinically available blood donor bag, hung in an incubator. 6,7,8,9) Each unit is attached to an actuator in a control system. The maximum magnitude of backpressure is regulated with a spring‐attached actuator for constant and cyclic HP. These culture units are completely closed and isolated from the ambient environment. Prior to reaching the set magnitude of HP, the HP/medium perfusion culture system automatically removed trapped air bubbles and periodically calibrated the HP with a reference of atmospheric pressure as zero. D) A photograph of HP/perfusion culture module. D) HP/perfusion culture system.

## MATERIALS AND METHODS

### Evaluation of the Performance of a Semipermeable Membrane Pouch

Prior to the experiments using cells, we evaluated the performance of a semipermeable membrane pouch at LOP (320 mOsm) and HOP (450 mOsm) supplemented with sodium chloride at 4.6 g/L.^25^ The OP was confirmed with a freeze point osmometer (Osmet, Precision, MA). We cut hollow fiber tubing made of polyvinylidene fluoride, 1 mm ID, 1.2 mm OD, and 500 KD cut‐off molecular weight (Spectrum Laboratories, Rancho Dominguez, CA) into pieces 35 mm in length.^26^ These pieces of tubing were immersed in 100% ethyl alcohol for 30 min, followed by Dullbecco's phosphate buffer saline (D‐PBS), and autoclaved at 121°C for 15 min. Each piece of tubing was filled with D‐PBS and sealed with stainless steel clips at both ends to fabricate a pouch. We suspended the pouches within a stainless‐steel mesh basket held in a 100 ml medium bottle and incubated in bovine serum albumin (Sigma–Aldrich) at 1.0 mg/ml with a stirrer at 5 spins/s to maintain enough mass transfer through a semipermeable membrane. Three pouches were collected after 24 and 48 h of incubation. Then, we collected a fluid sample from each pouch and measured the concentration of albumin with a BCA Protein assay kit (Pierce™, ThermoFisher Scientific, Waltham, MA). The performance of these pouches was evaluated with a ratio of albumin concentration inside and outside of each pouch with time.

### NP Cell/Cluster Isolation and Suspension Culture

We purchased fresh bovine tails (two‐ to three‐year‐old cows) from a local USDA‐certified slaughterhouse. We harvested caudal IVDs from the bovine tails using a blade (#15 and #22, BD), followed by the collection of NP tissues from each segment of IVD. Subsequently, we digested the NP tissues in 0.15% collagenase (Worthington, NJ) dissolved in Ham's F12 medium and sterilized with a 0.45 μm filter (Nalgene) at 37°C for 4 h on a rotator. Then we collected NP cells/clusters by centrifugation, rinsed them with D‐PBS twice, and seeded them onto 1.5% agarose‐coated six‐well plates. We incubated the NP cells/clusters in Dulbecco's Minimal Eagle Medium (DMEM)/Ham's F‐12 (1:1) with 10% fetal bovine serum and antibiotics for 3 to 4 days. During this incubation, NP cells/clusters were suspended in the culture medium, and the debris, for example, non‐digested matrix, was removed piece‐by‐piece with a pipette under a dissection microscope.

### Preparation of an NP Cell/Cluster Compartment Using a Semipermeable Membrane Pouch

To create semipermeable pouches, we injected a 1.0 × 10^5^ cell equivalent to the DNA value of NP cells/clusters into tubing and sealed the tubing with stainless steel clips to form pouches. We placed the pouches in a pressure‐proof chamber to load cyclic HP at 0–0.5 MPa, 0.5 Hz, medium replenishment at 0.1 ml/min, 3% O_2_ and 5% CO_2_ in air mimicking a physiological loading^27^ (Fig. 2).

### Incubation of NP Cells/Clusters With Various Combinations of Changes in HP And/or OP

The pouches were divided into three groups which had various combinations of no HP or HP (cyclic HP at 0.5 MPa, 0.5 Hz) and LOP or HOP as well as the order of these combinations were changed at day 4 (Fig. 1). These combinations were consolidated for each group and evaluated the interaction in the following studies. Group A: (A1) no HP/LOP for 7 days (control); (A2) HP/LOP; (A3) no HP/HOP; and (A4) HP/HOP; Group B: (B1) ascending HP/LOP; (B2) descending HP/LOP; (B3) ascending HP/HOP; (B4) descending HP/HOP; (B5) no HP/ascending OP, and B6) no HP/descending OP; Group C: (C1) simultaneously descending HP/OP and (C2) simultaneously ascending HP/OP. For no HP, we suspended the pouches within a stainless‐steel mesh basket held in a 100 ml medium bottle with a stirrer at 5 spins/s to maintain enough mass transfer through a semipermeable membrane.

### Evaluation of Gene Expression Using Quantitative‐Polymerase Chain Reaction (qPCR)

To analyze gene expression, we harvested the NP cells/clusters at day 7. The total RNA was extracted from the NP cells/clusters using an RNeasy kit® (Qiagen). The samples were homogenized in guanidine isothiocyanate based proprietary component of the kit with 1% β‐mercaptoethanol (RLT buffer) with a handheld homogenizer pestle (Fisher) in accordance with the manufacturer's protocol. The samples were amplified with a reverse transcriptase (High Capacity cDNA Reverse Transcription Kit, Life Technology). Gene expression master mix and fluorescent‐labeled specific primers (TaqMan®, Life Technology) were mixed, followed by quantitative‐PCR (7900HT, Applied Biosystems, Foster City, CA). The TaqMan® primers were a collagen type‐II, *COL‐2*: Bt03251837_mH; aggrecan core protein, *AGG*: Bt03212189_m1; proliferating cell nuclear antigen, *PCNA*: Bt03211154_g1; chondroitin sulfate N‐acetylgalactosaminyltransferase 1, *CSGALNACT1*: Bt03272948_m1, matrix metalloproteinase‐13, *MMP‐13*: Bt03214051_m1; and glyceraldehyde 3‐phosphate dehydrogenase, *GAPDH*: Hs03929097_g1 (Life Technology, Carlsbad, CA). Expression Suite Software v1.0.4 was used to analyze the data.

### Data Analysis of Gene Expression Profiles Using a qPCR Assay

Relative quantities (RQ) of the expression of each gene were calculated according to the difference between the average of each condition and of the no HP/LOP control (A1), which was given a value of 1.0. RQ was analyzed using a one‐way analysis of variance followed by a Bonferroni test to compare among all conditions with *p* < 0.05 considered significantly different (Stata, version 13; Statacorp LP, College Station, TX). The experiments were conducted five times. After outliers were eliminated with a Smirnov‐Grubbs test, statistical analyses of four or five samples were conducted.

### Evaluation of the Production of ECM Components Utilizing Immunohistology

We harvested cells/clusters by ejecting them with PBS. We then fixed them in a 2% paraformaldehyde/0.1 M cacodylate buffer (pH 7.4) at 4°C, embedded them in paraffin, and cut them into 7‐μm sections for immunostaining. Dewaxed sections were stained with a primary antibody against KS (1:500, Seikagaku, America), collagen type I (Col‐1, 1:100, Chemicon, Temecula, CA), collagen type II (Col‐2, 1:50, Chemicon, Temecula, CA), matrix mettaloproteinase‐13 (MMP‐13, 1:50, Santa Cruz Biotechnology, Santa Cruz, CA), and proliferating cell nuclear antigen (PCNA, 1:50, *NovoCastra*, New Castle, UK). Following the primary antibody, the sections were rinsed three times and incubated with a second biotinylated antibody according to the manufacturer's instructions (Vectastain™ ABC kit, Vector Laboratory). Color was developed with 3,38‐diaminobenzidine and nickel (DAB kit, Vector Laboratory). Counterstaining was performed with Harris's hematoxylin (Sigma‐Aldrich) for Col‐1, Col‐2, KS, and MMP‐13, and Contrast RED (KPL, Laboratories, Gaithersburg, MD) was used for PCNA.

## RESULTS

### Performance of the Semipermeable Membrane Pouch

**Figure 3:**
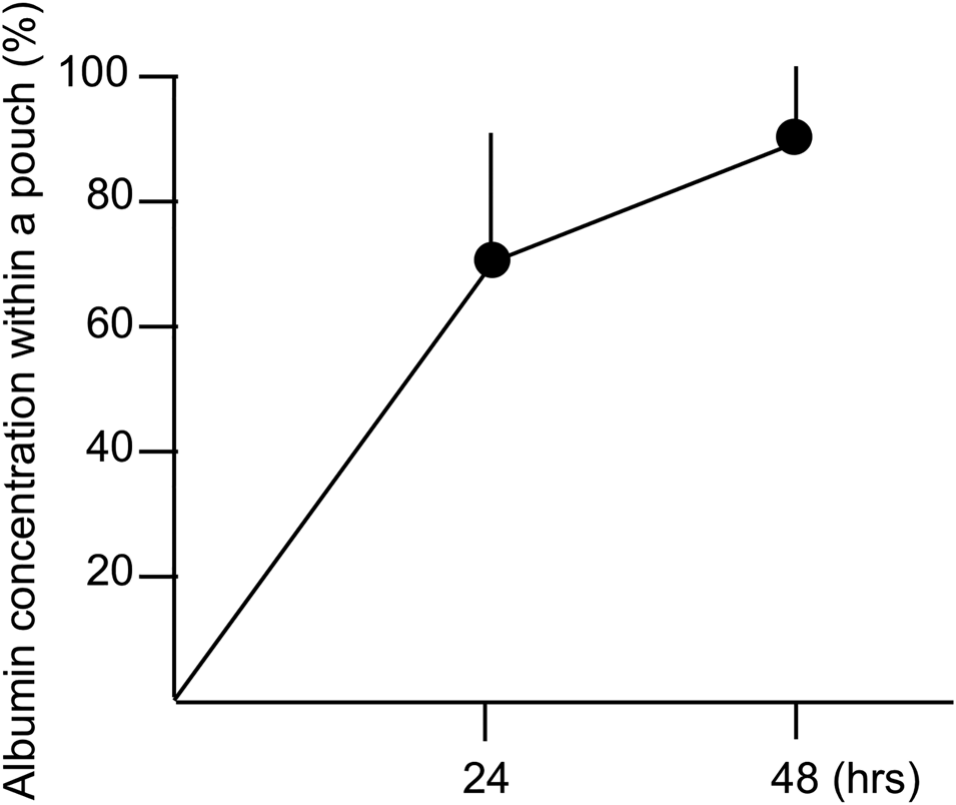
Performance of a semipermeable membrane pouch in a spinning culture vessel. Percentage of the concentration of bovine serum albumin of a solution within a pouch per albumin solution in a vessel.

We evaluated the performance of the semipermeable membrane pouch using BSA solution. The concentration of BSA within the pouch increased with time and resulted in 99.5% infiltration by 48 h after incubation (Fig. 3). This result allowed us to set the minimal duration of incubation for three days with the defined HP and OP conditions. Thus, further studies were conducted with combined HP and OP condition for the first 3 days of incubation, followed by a change to other conditions or a continuation of the same condition for the next 3 days.

### Gene Expression Profiles and Immunohistological Evaluation

**Figure 4:**
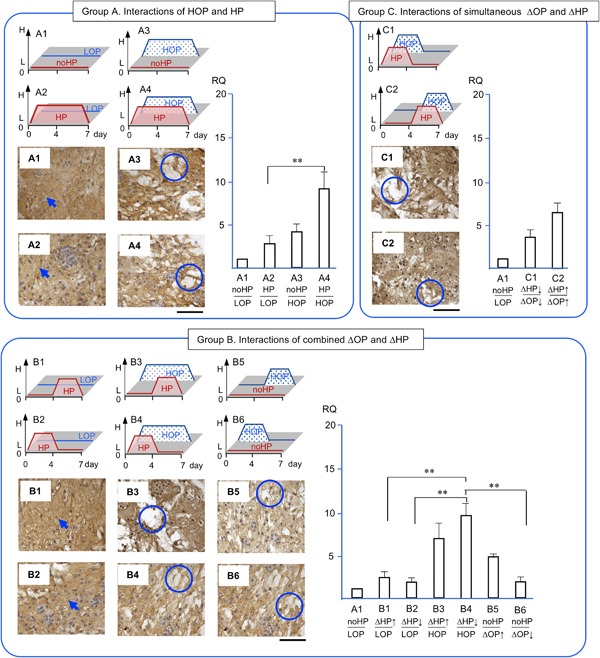
Gene expression profiles and immunohistology by NP cells/clusters in response to combined changes in hydrostatic pressure and osmotic pressure. The expression of aggrecan core protein (*AGG)* and the accumulation of sub‐molecule of aggrecan core protein, keratan sulfate in brown and counterstained with hematoxyline. RQ: relative quantity. Arrows indicate intense accumulation of keratan sulfate and circles indicate empty spaces. Each section is 7 μm thick and a bar indicate 100 μm. Mean ± SE (*n* = 5). **: *p* < 0.01.

We evaluated the gene expression of *AGG, COL‐2*, and *CSGALNACT1*, *MMP‐13*, and *PCNA* in NP cells/clusters in response to ΔHP and ΔOP grouped by counter combinations (Fig. 4). In addition, we histologically evaluated the quality of accumulated ECM, the localization of degenerative enzyme, and the capability of cell proliferation using antibodies and HABP (Fig. 4).

*AGG* expression was chosen as an anabolic marker of NP cells in response to combined ΔHP and ΔOP^25^, ^26^ (Fig. 4). In group A, *AGG* in HP/LOP (A2), no HP/HOP (A3), and HP/HOP (A4) showed a trend of 2.8, 4.1, and 9.1 times greater upregulation than the no HP/LOP control, respectively (A1). Particularly, HP/HOP (A4) upregulated *AGG* significantly 3.3 times greater than in HP/LOP (*p* < 0.05, A2). In group B, *AGG* in ΔHP/LOP (B1, B2) and no HP/descending OP (B6) were at a level similar to the control (A1). On the other hand, *AGG* in ascending HP/HOP (B3), descending HP/HOP (B4), and no HP/ascending OP (B5) showed a trend of 5.8, 9.5, and 4.7 times greater than the control (A1), respectively. Particularly, descending HP/HOP (B4) upregulated *AGG* significantly 4, 5.3, and 5.4 times greater than ascending HP/LOP (B1), descending HP/LOP (B2), and no HP/descending OP (B6), respectively (*p* < 0.01). In group C, *AGG* in simultaneously ascending HP/OP (C2) and descending HP/OP (C1) showed trends of 3.5 and 6.4 times greater upregulations than the control (A1), respectively.

KS was chosen to evaluate the quality of typical cartilaginous ECM, a component of aggrecan, in NP. KS showed mixture of fibrous and a dense area of ECM and intense pericellular matrix at each cell in all conditions (Fig. 4). A distinct difference was noted, namely, that a dense area of ECM was seen at LOP with HP and no HP (A1, A2, B1, B2). On the other hand, empty areas were seen in NP clusters at HOP with HP and no HP (A3, A4, B3, B4, B5, B6, C1, C2).

**Figure 5:**
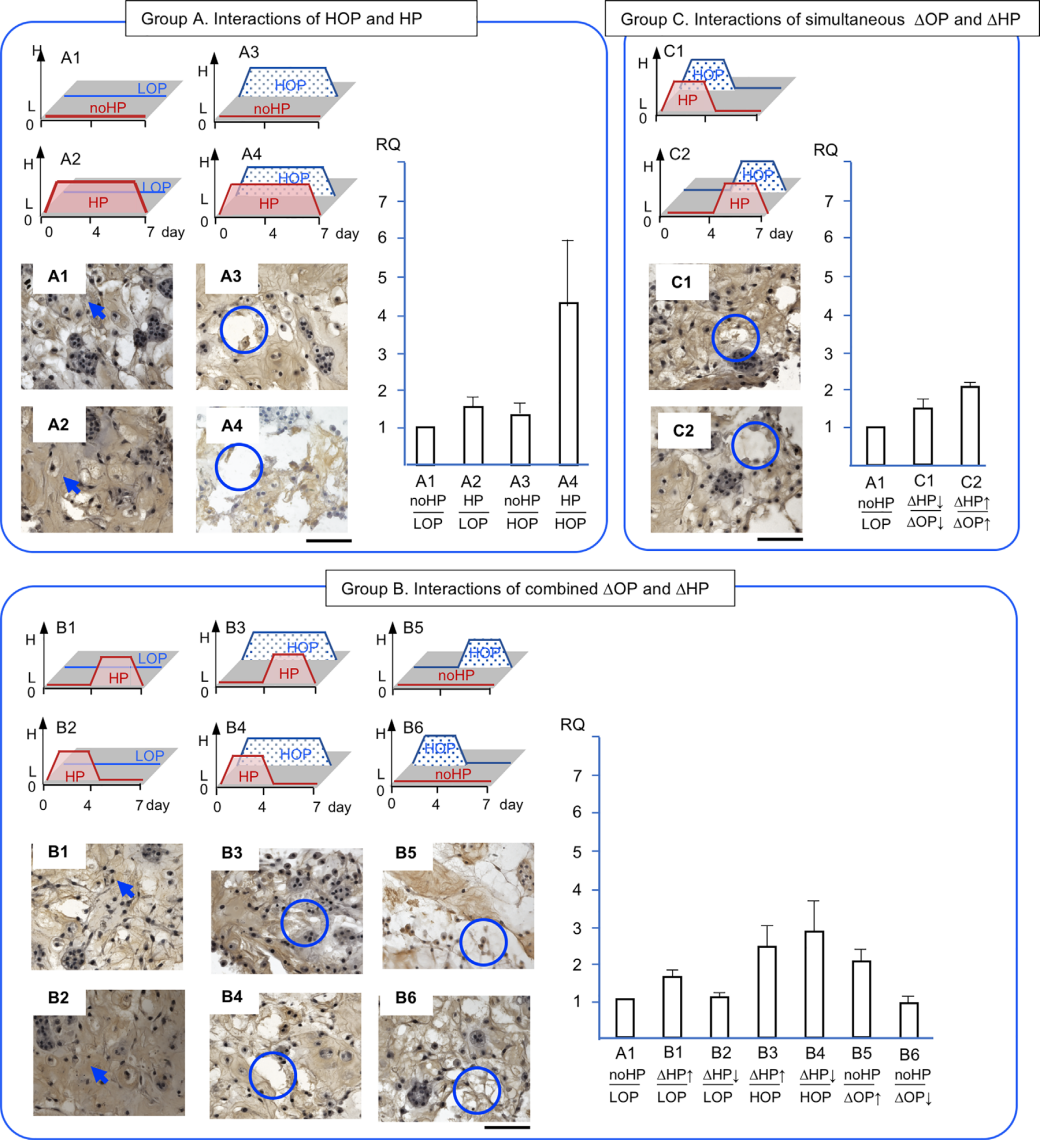
The expression of chondroitin sulfate N‐acerylgalactosaminyltransferase (*CSGLUNACT*) and the accumulation of hyaluronan binding protein (HABP) in brown and counterstained with hematoxyline. RQ: relative quantity. Arrows indicate intense accumulation of HABP and circles indicate empty spaces. Each section is 7 μm thick and a bar indicate 100 μm. Mean ± SE (*n* = 5).

*CSGALNACT1* expression was chosen as an anabolic marker and essential enzyme to synthesize GAG^28^, ^29^ (Fig. 5). In group A, *CSGALNACT1* in HP/LOP (A2) and no HP/HOP (A3) were at a level similar to *CSGALNACT1* in no HP/LOP control (A1). *CSGALNACT1 in* HP/HOP (A4), however, showed a trend of 4.3 times greater upregulation than the control (A1). In group B, *CSGALNACT1* in ascending and descending HP/HOP (B3, B4) showed a trend of 2.4 and 2.7 times greater upregulations than the control (A1), respectively. In group C, *CSGALNACT1* in simultaneously descending HP/OP (C1) and ascending HP/OP (C2) were at a level of the expression similar to in the control (A1), respectively.

HABP specifically enabled the binding of hyaluronan and aggrecan core‐protein^30^ (Fig. 5). Thus, this molecule is supposed to co‐localize with chondroitin sulfate proteoglycan. The dense HABP was seen at HP/LOP and no HP/LOP (A1, A2, B1, B2) than at no HP/HOP and HP/HOP (A3, A4, B3, B4, B5, B6, C1, C2).

**Figure 6:**
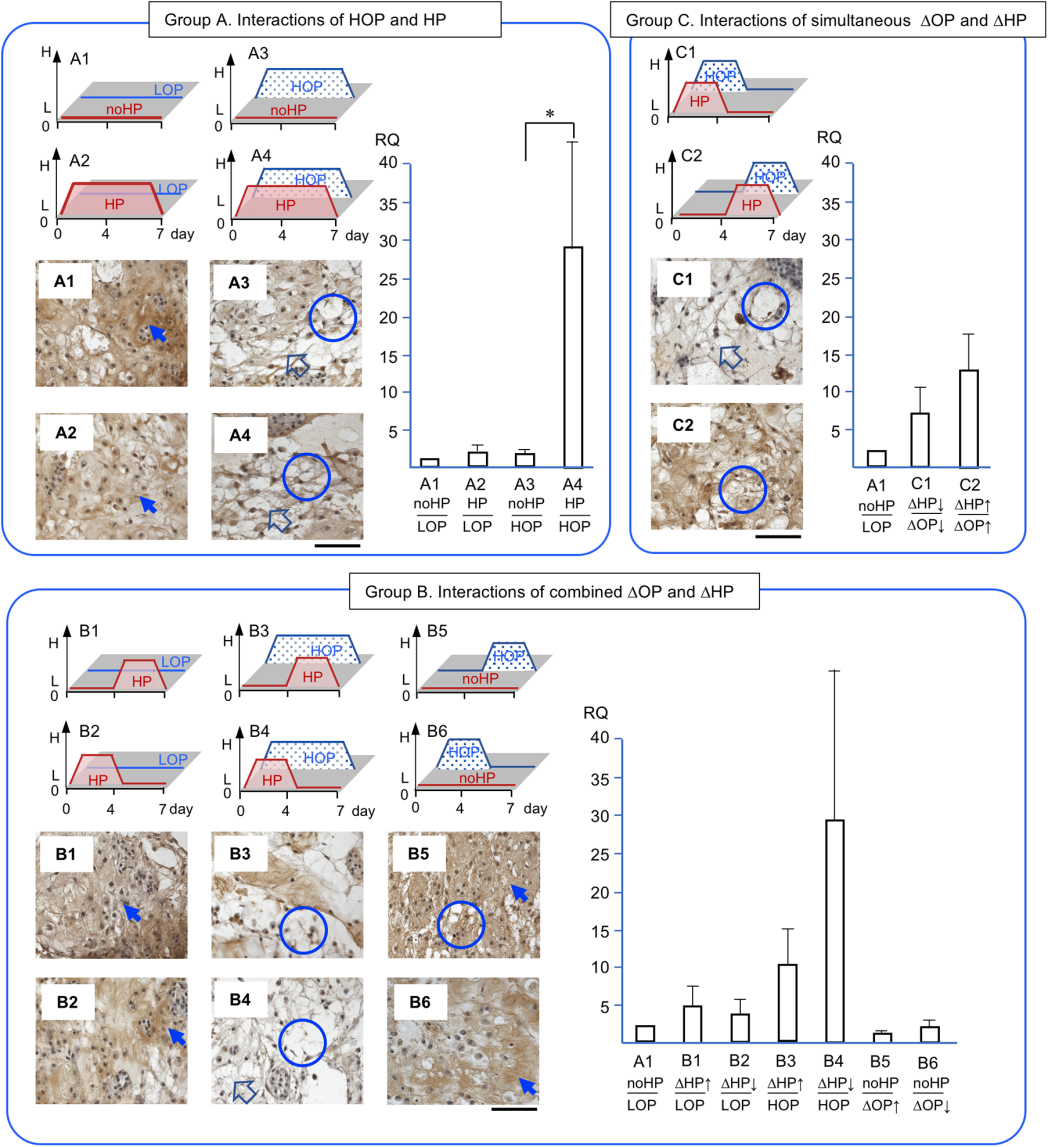
The expression of collagen type‐2 (*COL‐2*) and the accumulation of Col‐2 in brown and counterstained with hematoxyline. RQ: relative quantity. Arrows indicate intense accumulation of COL‐2 and circles indicate empty spaces. Each section is 7 μm thick and a bar indicate 100 μm. Mean ± SE (*n* = 5). *: *p* < 0.05.

*COL‐2* expression was chosen as an anabolic marker^1^ (Fig. 6). In group A, *COL‐2* in HP/HOP (A4) upregulated significantly 17.3 times greater than in no HP/HOP (A3) (*p* < 0.05). In group B, *COL‐2* in ascending HP/LOP (B1) was at a level similar to descending HP/LOP (B2). However, *COL‐2* in ascending HP/HOP (B3) and descending HP/HOP (B4) were 10 and 30 times greater than in the control (A1), respectively. In group C, *COL‐2* in simultaneously descending HP/OP (C1) and simultaneously ascending HP/OP (C2) were 7.0 and 12.8 times greater than in the control (A1), respectively.

COL‐2 was chosen to evaluate the quality of typical cartilaginous ECM in NP (Fig. 6). COL‐2 showed mixture of fibrous and a dense area of ECM in LOP with no HP and HP (A1, A2, B1, B2). COL‐2 with short exposure (3 days) to HOP showed lesser density of accumulation (B5, B6, C1, C2). In addition, COL‐2 with longer exposure (7 days) showed empty spaces and fibrous ECM (A3, A4, B3, B4).

**Figure 7:**
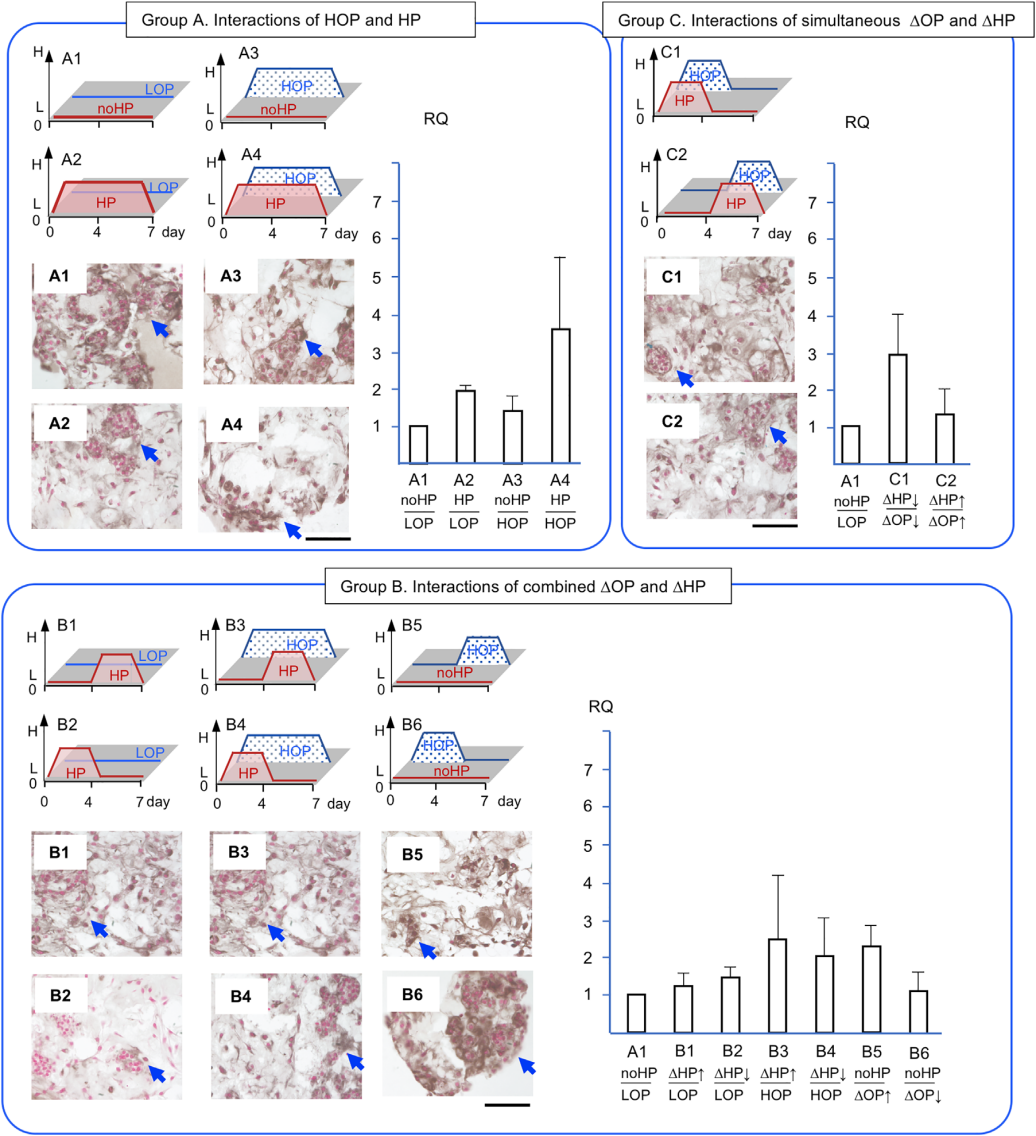
The expression of matrix metalloproteinase‐13 (*MMP‐13*) and the accumulation of MMP‐13 in black and counterstained with a contrast red in red. RQ: relative quantity. Arrows indicate intense accumulation of MMP‐13. Each section is 7 μm thick and a bar indicate 100 μm. Mean ± SE (*n* = 5).

*MMP‐13* expression was chosen as a catabolic marker^31^ (Fig. 7). In group A, *MMP‐13* in HP/LOP (A2) and HP/HOP (A4) showed a trend of 2 and 3.5 times greater upregulation, respectively, than in no HP/LOP control (A1). In group B, *MMP‐13* in ascending HP/LOP (B1) and descending HP/LOP (B2) was at a level similar to the control (A1). On the other hand, ascending HP/HOP (B3), descending HP/HOP (B4), and no HP/ascending OP (B5) showed a trend of 2.5, 2.0, and 2.3 times greater upregulation than the control (A1), respectively. In group C, *MMP‐13* in simultaneously descending HP/OP (C1) showed a trend of 2.9 times greater upregulation than the control (A1), and simultaneously ascending HP/OP (C2) was at a level similar to the control (A1).

MMP‐13 was chosen to locate the degeneration of collagen type I, II, and III (Fig. 7). Since the amount of this enzyme was expected to be low, we use a high contrast chromophore in black and counterstained in red. Distinctively stained MMP‐13 was seen around cell clusters compared to the ECM area in the NP clusters. Distinctive differences of locations among culture conditions, however, were not seen.

**Figure 8:**
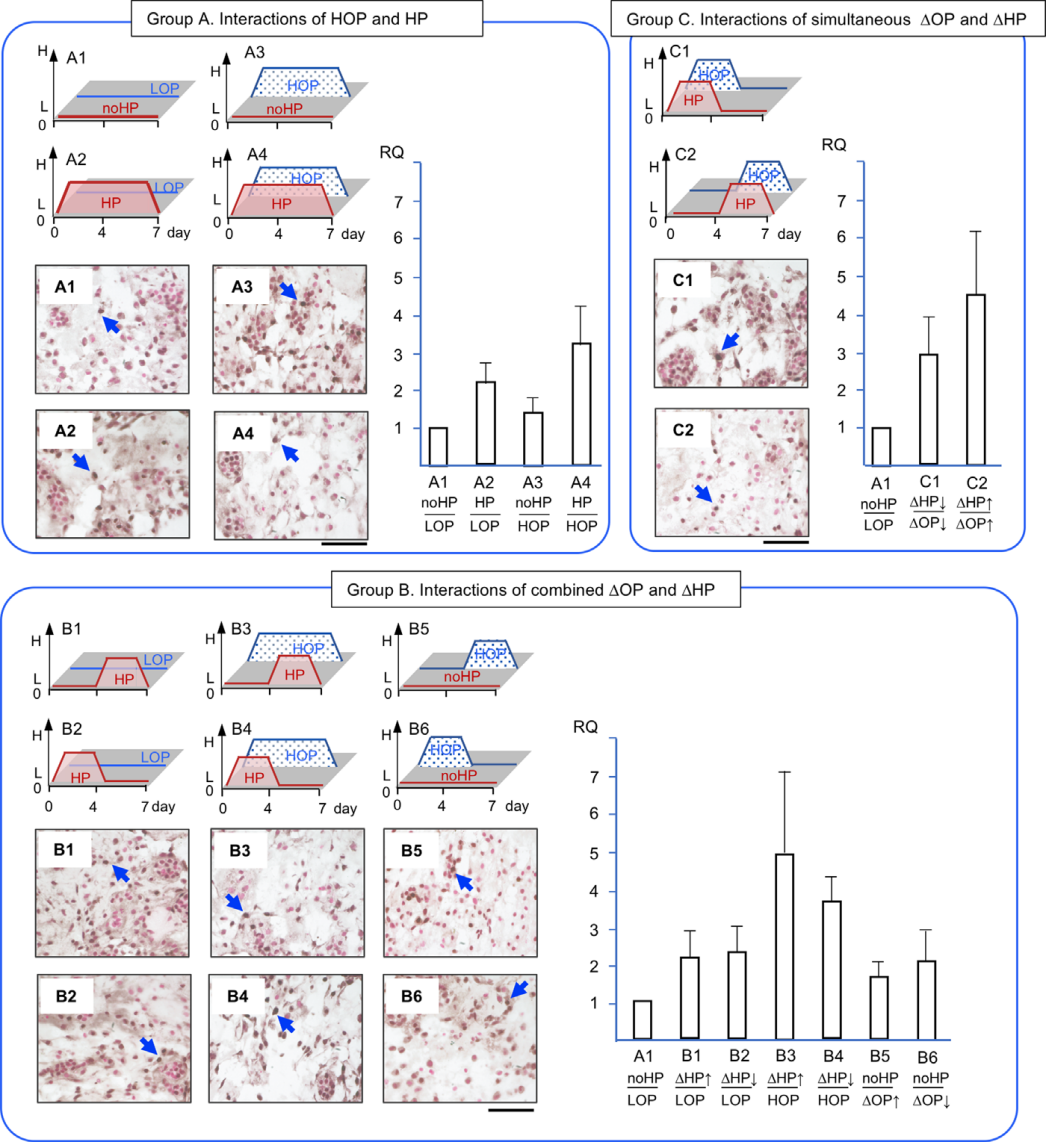
The expression of proliferating cell nuclear antigen (*PCNA*) and the accumulation of PCNA in black and counterstained with a contrast red in red. RQ: relative quantity. Arrows indicate a PCNA positive cell. Each section is 7 μm thick and a bar indicate 100 μm. Mean ± SE (*n* = 5).

*PCNA* expression was chosen as a cell proliferation marker (Fig. 8). In order to determine homeostasis and regenerative capability, it was important to know that changes in HP and/or OP stimulate NP cell proliferation. In group A, *PCNA* in HP/HOP (A4) showed a trend of 3.2 times greater upregulation than no HP/LOP control (A1). In group B, *PCNA* in ascending HP/HOP (B3) and descending HP/HOP (B4) showed a trend of 4.8 and 3.6 times greater upregulation than in the control (A1), respectively. In group C, *PCNA* in simultaneously descending HP/OP (C1) and ascending HP/OP (C2) showed a trend of 2.8 and 4.5 times greater upregulation than in the control (A1), respectively.

PCNA positive cells were frequently seen within the NP cell clusters (Fig. 8). Each photomicrograph included images of these clusters and distributions of individual cells within one frame. Although multiple samples were stained with antibody against PCNA, distinct differences as the number of positive cells among the culture conditions were not noted. However, among the locations, the number of PCNA positive cells within the cell clusters was greater than within the area of ECM.

## DISCUSSION

Our ultimate goal was to clarify the roles of NP cells and ECM in homeostasis, degeneration, and regeneration of NP tissue when changes in physicochemical stresses occur. To this end, we suspended amorphous NP cells/clusters within a compartment of a semipermeable membrane pouch and incubated them with various combinations of ΔHP and ΔOP in vitro.

### Justification of Methodology

The pouches allowed the cells/clusters to stay alive and the ECM to accumulate within them.^25^ The concentration of infiltrated serum albumin in the pouch was equivalent to the concentration of albumin in the solution outside of the pouch within 48 h (Fig. 3). Therefore, we determined that this pouch allowed a sufficient mass transfer of nutrients and gases to maintain cellular activity. Another advantage of using the pouch was that NP cells was not influenced by the fluid shear stress of medium. Although molecules smaller than albumin could infiltrate the pouch faster, we think that, overall, biological molecules were infiltrated within a reasonable duration. Thus, a pouch device can be used for amorphous cells/clusters construct.

We realized that bovine caudal NP tissue had distinct cell populations: Individual and cluster.^32^, ^33^ Therefore, we wanted to maintain the cell‐ECM and cluster‐ECM connections of both of these populations seen in native NP. We isolated NP cells/clusters enzymatically from bovine caudal NPs and incubated them on non‐adherent culture substrate (coated with agarose gel) prior to the reconstitution of NPs using a semipermeable membrane pouch. This method allowed for the removal of large debris from the cells/cluster suspension piece‐by‐piece using a pipette and of small fragments, including erythrocytes, without centrifugation, or filtration. In this typical cell suspension, the NP cells were sparse within ECM, and cell clusters of 5 to 15 cells were retained. It would have been difficult to handle the cells/clusters due to their fragility. But the use of a semipermeable membrane pouch made it easy to handle amorphous NP cells/clusters securely.

We chose *AGG* and *COL‐2* as anabolic biomarkers and *CSGLUNACT1* as an enzyme necessary to synthesize glycosaminoglycan because the NP cells produced an abundant amount of aggrecan and collagen type II.^1^, ^28^, ^29^, ^30^, ^31^ These gene expressions were supported by immunohistological evidence of corresponding antibodies and HABP.^34^ In addition, the frequency of cell proliferation and location trends were determined with *PCNA* and its antibody. We chose *MMP‐13* and its antibody to determine catabolic activity and its location within NP cells/clusters.^31^ With these markers above, we evaluated the interactions of combined ΔHP and ΔOP over 7 days. We compared the RQ of each condition with LHP/LOP (A1) as a control. Although the changes in the stresses repetitively occurred, we wanted to compare data within the shortest duration, but covering relevant combinations of changes in HP and OP.

We conducted this study with the magnitude of HP at 0.5 MPa. This magnitude was within equivalent ranges of intradiscal pressure at various spinal positions in humans.^27^ Although in vivo intradiscal pressure and in vitro HP in culture medium are independently discussed, we thought that the magnitude of HP in our study was approximate to the physiological level of pressure in vivo. The frequency of HP in this study was set at 0.5 Hz to allow necessary mass transfer to NP cells/clusters enclosed within the semipermeable membrane pouch.^25^

We used sodium chloride to alter OP in culture. OP in the IVD was determined by conversion from fixed charged density and estimated at 450–540 mOsm.^14^, ^20^ It is unclear whether OP in cell culture medium really mimics OP in native NP. In recent studies, investigators have used a supplemented medium (e.g., polyethylene glycol) to alter OP.^14^ However, this supplemented medium has not resolved the theoretical differences between medium and native NP. In addition, changing OP with an ion‐based solute may cause changes in the volume of a cell and subsequently alter subcellular structural and ECM‐associated signal molecules, for example, integrin,^35^ as well as ion channels.^36^, ^37^ Thus, we think that involvements of partitioning NP tissue causing intrinsic swelling pressure are missing properties in this NP cells/clusters model.

### The Effects of Changes in Physicochemical Stresses on NP Cells and ECM

We consolidated the 12 conditions of combined ΔHP and ΔOP into three groups to reproduce the various combined physicochemical stresses that occur in native NP (Fig. 4, 5, 6, 7, 8). Our first objective was to clarify the interactions of HP and OP over 7 days. The data representing these interactions were obtained from A1, 2, 3, and 4. During the 7 days of exposure to HP/HOP (A4), all genes were upregulated; however, ECM had empty spaces. Thus, the level of gene expression of ECM molecules and the quality of accumulated ECM seemed inconsistent. Particularly, the inconsistency was noted when NP cells were incubated in HOP medium. These results were likely the product of undetermined catabolic molecules and/or the cause of osmotic imbalance in ECM. Since aggrecan had fixed charged density of chondroitin sulfate, it was possible to attract ions and absorb water, resulting in increasing a repulsion of ECM. In our other study, we could see similar empty spaces in ECM within a chondrocyte spheroid model at HOP.^43^ Thus, we think that degeneration of ECM was not only a cell‐specific event but physicochemical properties of ECM. Meanwhile, these NP cells/clusters may represent an anabolic phase because they started ECM accumulation after seeding enzymatically isolated cells. We speculated that the cells/clusters could optimize an accumulation of the newly synthesized ECM at LOP environment resulted in dense ECM.

Second, we wanted to clarify the effects of ΔHP at HOP or LOP. The data representing these effects were obtained from B1–B6 (Fig. 4, 5, 6, 7, 8). The ΔHP did not impact the level of gene expression at LOP, whereas ΔHP/HOP had significantly stimulated *AGG* expressions and showed a trend of greater upregulations of other anabolic genes. Physiologically, these conditions were thought to occur during our normal spinal motion. Therefore, these results strongly support our hypothesis for homeostasis or regeneration in NP at gene expression level. We speculate that repetitive ΔHP at HOP is effective to regenerate NP if we could minimize degeneration of ECM. We will seek culture conditions allowing denser accumulation of ECM in further studies.

Third, we wanted to clarify the interactions of simultaneously ΔHP and ΔOP. The data representing these interactions were obtained from C1 and C2 (Fig. 4, 5, 6, 7, 8). We thought that these conditions did not occur during normal daily spinal motion. Thus, we thought these conditions mimicked traumatic physicochemical changes in NP. Simultaneously descending HP and OP (C1) showed a trend of higher *MMP‐13* expression than simultaneously ascending HP and OP (C2). In addition, degenerative sign, a granule, of COL‐2 was seen in the former condition.^38^, ^39^ Thus, we think that these results strongly support our hypothesis for traumatic degeneration in NP at gene expression level and with histological characteristics.

### Relevance of the NP Cell/Cluster Model With ΔHP and ΔOP

Using MRI T2 mapping, we clinically determined that degenerative disc had less amount of water compared to a healthy disc.^40^, ^41^ Thus, one can translate that pathogenesis of a degenerative NP leads to a loss of interstitial water. If the empty space in ECM seen at HOP was filled with water, it was not explanation to determine degenerative characteristics. In this study, we had limitations to determine whether empty spaces were created with infiltrated water or resulted in repulsion of ECM. The NP cells/clusters were suspended within a semipermeable membrane pouch, which was an unconfined compartment. If the clusters were confined at a certain level, we speculate that the clusters generate a swelling pressure in the pouch compartment from newly accumulated synthesized ECM. We think that further studies will include the aspect of swelling pressure on interactions of physicochemical stresses in NP.

Another inconsistency of immunohistology in the MMP‐13 stain was found. There was lesser gene expression of *MMP‐13* at LOP, but the histology showed more positive MMP‐13. We think that MMP‐13‐positive ECM at HOP disappeared and less damaged ECM could retain MMP‐13.

The justification for the duration, minimum 3 days, of each condition was determined based on our other studies using bovine and human articular chondrocytes because this duration allowed us to detect RQ of qPCR.^25^, ^42^ In order to maximize gene expression of anabolic molecules and reduce the empty space in ECM for the regenerative treatment strategy, we think that fewer than 3 days of HP/HOP (A4, B3, B4) followed by no HP/LOP (A1) and/or HP/LOP (A4) repetitively would be a potential condition to gain anabolic gene expression and ECM accumulation in NP cells/clusters in vitro. This condition shall be examined in further studies.

## CONCLUSION

The convenient cell cultures are all being conducted under LOP and LHP (A1). Our results would suggest that adding HP and HOP would have a significant impact on matrix homeostasis by NP cells. Therefore, the results of the convenient cell cultures may not represent what is happening in vivo. We systematically evaluated a set of anabolic, catabolic, and cellular characteristics of NP cells in response to ΔHP and ΔOP. Structural retention of ECM is key to reproducing consistent gene expression and histology in NP cells/clusters. The foundation that this study lays will allow us to move toward future mechanistic studies on intracellular mechanosignal transduction, which mediates these responses to physicochemical stresses. These studies will provide new insights into the effects of ΔHP/ΔOP on matrix homeostasis and allow for improved biological therapeutic strategies to treat intervertebral disc degeneration.

## AUTHORS’ CONTRIBUTION

SM and JDK designed the experiments and analyzed the data including statistical analysis and immunohistology. KK contributed to sample preparations. SM prepared the manuscript, and all authors contributed and provided critical feedback. SM conducted all experiments, gave the final approval for the submitted version of the manuscript.

## References

[1] Hayes AJ , Benjamin M , Ralphs JR . 2001 Extracellular matrix in development of the intervertebral disc. Matrix Biol 20:107–121. 11334712 10.1016/s0945-053x(01)00125-1

[2] Urban JP , Roberts S . 2003 Degeneration of the intervertebral disc. Arthritis Res Ther 5:120–130. 12723977 10.1186/ar629 PMC165040

[3] Setton LA , Chen J . 2006 Mechanobiology of the intervertebral disc and relevance to disc degeneration. J Bone Joint Surg 88:52–57. 16595444 10.2106/JBJS.F.00001

[4] Vergroesen P‐PA , van der Veen AJ , van Royen BJ , et al. 2014 Intradiscal pressure depends on recent loading and correlates with disc height and compressive stiffness. Eur Spine J 23:2359–2368. 25031105 10.1007/s00586-014-3450-4

[5] Wuertz K , Urban JPG , Klasen J , et al. 2007 Influence of extracellular osmolarity and mechanical stimulation on gene expression of intervertebral disc cells. J Orthop Res 25:1513–1522. 17568421 10.1002/jor.20436

[6] Schroeder Y , Sivan S , Wilson W , et al. 2007 Are disc pressure, stress, and osmolarity affected by intra‐ and extrafibrillar fluid exchange? J Orthop Res 25:1317–1324. 17557324 10.1002/jor.20443

[7] Urban JP , McMullin JF . 1998 Swelling pressure of the lumbar intervertebral discs: influence of age, spinal level, composition, and degeneration. Spine 13:179–187. 10.1097/00007632-198802000-00009 3406838

[8] Sivan SS , Wachtel E , Roughley P . 2014 Structure, function, aging and turnover of aggrecan in the intervertebral disc. Biochimica Biophysica Acta 1840:3181–3189. 10.1016/j.bbagen.2014.07.013 25065289

[9] Schmidt H , Shirazi‐Adl A , Schilling C , et al. 2016 Preload substantially influences the intervertebral disc stiffness in loading‐unloading cycles of compression. J Biomech 49:1926–1932. 27209550 10.1016/j.jbiomech.2016.05.006

[10] Chan SCW , Walser J , Ferguson SJ , et al. 2015 Duration‐dependent influence of dynamic torsion on the intervertebral disc: an intact disc organ culture study. Eur Spine J 24:2402–2410. 26215177 10.1007/s00586-015-4140-6

[11] O'Connell GD , Vresilovic EJ , Elliott DM . 2011 Human intervertebral disc internal strain in compression: the effect of disc region, loading position, and degeneration. J Orthop Res 29:547–555. 21337394 10.1002/jor.21232 PMC3428014

[12] Neidlinger‐Wilke C , Mietsch A , Rinkler C , et al. 2012 Interactions of environmental conditions and mechanical loads have influence on matrix turnover by nucleus pulposus cells. J Orthop Res 30:112–121. 21674606 10.1002/jor.21481

[13] Dijk BGM , Potier E , Ito K . 2013 Long‐term culture of bovine nucleus pulposus explants in a native environment. Spine J 13:454–463. 23340344 10.1016/j.spinee.2012.12.006

[14] Spillekom S , Smolders LA , Grinwis GCM , et al. 2014 Increased osmolarity and cell clustering preserve canine notochordal cell phenotype in culture. Tissue Eng Part C 20:652–662. 10.1089/ten.TEC.2013.0479 24304309

[15] Roberts S , Menage J , Sivan S , et al. 2008 Bovine explant model of degeneration of the intervertebral disc. BMC Musculoskelet Disord 9:24. 18298830 10.1186/1471-2474-9-24 PMC2266744

[16] Hartman RA , Bell KM , Debski RE , et al. 2012 Novel ex‐vivo mechanobiological intervertebral disc culture system. J Biomech 45:382–385. 22099147 10.1016/j.jbiomech.2011.10.036 PMC3246121

[17] Tsai TL , Nelson B , Anderson PA , et al. 2014 Intervertebral disc and stem cells cocultured in biomimetic extracellular matrix stimulated by cyclic compression in perfusion bioreactor. Spine J 14:2127–2140. 24882152 10.1016/j.spinee.2013.11.062

[18] Junger S , Gantenbein‐Ritter B , Lezuo P . 2009 Effects of limited nutrition on in situ intervertebral disc cells under simulated‐physiological loading. Spine 34:1264–1271. 19455001 10.1097/BRS.0b013e3181a0193d

[19] Lee CR , Iatridis JC , Poveda L , et al. 2006 *In vitro* organ culture of the bovine intervertebral disc. Spine 31:515–522. 16508544 10.1097/01.brs.0000201302.59050.72 PMC7187957

[20] Wuertz K , Urban JPG , Klasen J , et al. 2007 Influence of extracellular osmolarity and mechanical stimulation on gene expression of intervertebral disc. J Orthop Res 25:1513–1522. 17568421 10.1002/jor.20436

[21] Bibby SR , Jones DA , Ripley RM , et al. 2005 Metabolism of the intervertebral disc: effects of low levels of oxygen, glucose, and pH on rates of energy metabolism of bovine nucleus pulposus cells. Spine 30:487–496. 15738779 10.1097/01.brs.0000154619.38122.47

[22] Maroudas A , Stockwell RA , Nachemson A , et al. 1975 Factors involved in the nutrition of the human lumbar intervertebral disc: cellularity and diffusion of glucose in vitro. J Anat 120:113–130. 1184452 PMC1231728

[23] Urban JP , McMullin JF . 1998 Swelling pressure of the lumbar intervertebral discs: influence of age, spinal level, composition, and degeneration. Spine 13:179–187. 10.1097/00007632-198802000-00009 3406838

[24] Setton LA , Chen J . 2004 Cell mechanics and mechanobiology in the intervertebral disc. Spine 29:2710–2723. 15564920 10.1097/01.brs.0000146050.57722.2a

[25] Mizuno S , Ogawa R . 2011 Using changes in hydrostatic and osmotic pressure to manipulate metabolic function in chondrocytes. Am J Physiol‐Cell Physiol 300:C1234–C1245. 21270297 10.1152/ajpcell.00309.2010

[26] Takada E , Mizuno S . 2018 Reproduction of characteristics of extracellular matrices in specific longitudinal depth zone cartilage within spherical organoids in response to changes in osmotic pressure. Int J Mol Sci 19:1507–1517. 10.3390/ijms19051507 PMC5983583 29783650

[27] Wilke HJ , Neef P , Caimi M , et al. 1999 New in vivo measurement of pressures in the intervertebral disc in daily life. Spine 24:755–762. 10222525 10.1097/00007632-199904150-00005

[28] Iatridis JC , MacLean JJ , O'Brien M , et al. 2007 Measurements of proteoglycan and water content distribution in human lumbar intervertebral discs. Spine 32:1493–1497. 17572617 10.1097/BRS.0b013e318067dd3f PMC3466481

[29] Sztrolovics R , Alini M , Roughley PJ , et al. 1997 Aggrecan degradation in human intervertebral disc and articular cartilage. Biochem J 326:235–241. 9337874 10.1042/bj3260235 PMC1218660

[30] Scott JE , Bosworth TR , Cribb AM , et al. 1994 The chemical morphology of age‐related changes in human intervertebral disc glycosaminoglycans from cervical, thoracic and lumbar nucleus pulposus and annulus fibrosus. J Anat 184:73–82. 8157495 PMC1259928

[31] Sakai K , Kimata K , Sato T , et al. 2007 Chondroitin sulfate N‐acetylgalactosaminyltransferase‐1 plays a critical role in chondroitin sulfate synthesis in cartilage. J Biol Chem 282:4152–4161. 17145758 10.1074/jbc.M606870200

[32] Johnson WE , Eisenstein SM , Roberts S . 2001 Cell cluster formation in degenerate lumbar intervertebral discs is associated with increased disc cell proliferation. Connect Tissue Res 42:197–207. 11913491 10.3109/03008200109005650

[33] Hunter CJ , Matyas JR , Duncan NA . 2004 The functional significance of cell clusters in the notochordal nucleus pulposus. Spine 29:1099–1104. 15131437 10.1097/00007632-200405150-00010

[34] Tengblad A , Pearce RH , Grimmer BJ . 1984 Demonstration of link protein in proteoglycan aggregates from human intervertebral disc. Biochem J 222:85–92. 6477515 10.1042/bj2220085 PMC1144147

[35] Jablonski CL , Ferguson S , Pozzi A , et al. 2014 Integrin a1b1 participates in chondrocyte transduction of osmotic stress. Biochem Biophys Res Commun. 445:184–190. 24495803 10.1016/j.bbrc.2014.01.157 PMC4022045

[36] Mavrogonatou E , Kletsas D . 2010 Effect of varying osmotic conditions on the response of bovine nucleus pulposus cells to growth factors and the activation of the ERK and Akt pathways. J Orthop Res 28:1276–1282. 20309957 10.1002/jor.21140

[37] Johnson ZI , Shapiro IM , Risbud MV . 2014 Extracellular osmolarity regulates matrix homeostasis in the intervertebral disc and articular cartilage: evolving role of TonEBP. Matrix Biol 40:10–16. 25172826 10.1016/j.matbio.2014.08.014 PMC4390124

[38] Korecki CL , Kuo CK , Tuan RS , et al. 2009 Intervertebral disc cell response to dynamic compression is age and frequency dependent. J Orthop Res 27:800–806. 19058142 10.1002/jor.20814 PMC2757142

[39] Adams MA , McNally DS , Dolan P . 1996 'Stress' distributions inside intervertebral discs. The effects of age and degeneration. J Bone Joint Surg Br 78:965–972. 8951017 10.1302/0301-620x78b6.1287

[40] Sowa G , Vadalà G , Studer R , et al. 2008 Characterization of intervertebral disc aging: longitudinal analysis of a rabbit model by magnetic resonance imaging, histology, and gene expression. Spine 33:1821–1828. 18670334 10.1097/BRS.0b013e31817e2ce3

[41] Ellingson AM , Nagel TM , Polly DW , et al. 2014 Quantitative T2* (T2 star) relaxiation times predict site specific proteoglycan content and residual mechanics of the intervertebral disc throughout degeneration. J Orthop Res 32:1083–1089. 24788830 10.1002/jor.22633 PMC4136382

[42] Ogura T , Tsuchiya A , Minas T , et al. 2017 Optimization of extracellular synthesis and accumulation by human articular chondrocytes in 3‐dimentional construct with repetitive hydrostatic pressure. Cartilage. DOI: 10.1177/1947603517743546. PMC5871128 29262701

[43] Iatridis JC , Godburn K , Wuertz K , et al. 2011 Region‐dependent aggrecan degradation patterns in the rat intervertebral disc are affected by mechanical loading in vivo. Spine 36:203–209. 20714280 10.1097/BRS.0b013e3181cec247 PMC2988868

